# Severe Gastroparesis following Radiofrequency Catheter Ablation for Atrial Fibrillation: Suggestion for Diagnosis, Treatment, and Device for Gastroparesis after RFCA

**DOI:** 10.1155/2014/923637

**Published:** 2014-12-30

**Authors:** Dong Seok Lee, Sang Jin Lee

**Affiliations:** ^1^Department of Internal Medicine, Gangneung Asan Medical Center, University of Ulsan College of Medicine, Gangneung, Republic of Korea; ^2^Department of Internal Medicine, Gangneung Asan Medical Center, Sacheon-myeon, Gangneung, Gangwon-do 210-711, Republic of Korea

## Abstract

Gastroparesis following radiofrequency catheter ablation (RFCA) is a very rare complication, as only two cases have been reported in the English literature. A 42-year-old man underwent RFCA due to recurrent drug-resistant symptomatic atrial fibrillation. The patient complained of indigestion and early satiety 2 days after the procedure. Contrast-enhanced computed tomography and an upper gastrointestinal series of the abdomen showed a large amount of material remaining in the stomach area. All food material was removed by endoscopy, and the patient received medical treatment. We suggest a flow chart for diagnosis and treatment of AFGS based on the present case and previous cases. Endoscopic medical patent was designed on the basis of this case.

## 1. Introduction

The definition of gastroparesis is a delay in emptying of food from the stomach. The causes of gastroparesis are postvagotomy syndrome of a peptic ulcer, pancreatic adenocarcinoma, mesenteric vascular insufficiency, and systemic disease such as amyloidosis. Most patients with gastroparesis complain of abdominal distension and epigastric discomfort after eating. No ulcers or gastric outlet obstructions are found on an upper endoscopy exam. A gastrointestinal motility study is widely conducted to diagnose gastroparesis.

The radiofrequency catheter ablation (RFCA) procedure is widely performed to treat atrial fibrillation (AF). However, various complications have been reported. Thromboembolic events, tamponade, severe pulmonary vein stenosis, femoral pseudoaneurysm, and atrioesophageal fistula have been reported as complications of the RFCA procedure [1–3]. A few cases of vagus nerve injury due to RF ablation energy have been reported and retrospective studies have been conducted [4–6].

## 2. Case Report

A 59-year-old man presented with a history of paroxysmal palpitation for more than a few years. Symptom duration and frequency had been prolonged recently with a documented ECG. He was treated with digoxin, quinidine, propafenone, metoprolol, and amiodarone sequentially. However, his symptoms persisted. He was diagnosed with drug-refractory, symptomatic, chronic AF and underwent RFCA. The patient was transferred to the EP laboratory. After an esophagram, we positioned the catheters and performed a dual septal puncture. The baseline ECG indicated normal sinus rhythm. Ablation at the left superior pulmonary vein (LSPV) was started and electrical isolation of the LSPV, left inferior pulmonary vein, right superior pulmonary vein, and right inferior pulmonary vein as well as a cavotricuspid isthmus block was completed. There was atrial flutter (AFL) on the left atrial side. An activation map was prepared. The AFL went through the roof and the perimitral area. We induced AF with ISO 5. After RFCA, he was admitted to the intensive care unit due to the long procedure time, 7 hours. No immediate complications were detected during the procedure. Sinus rhythm was maintained after the procedure. Patient has no underlying disease except for arrhythmia and did not complain of anxiety every day, before and after RFCA. But he complained of abdominal distension and epigastric discomfort after eating on hospital day 2. Bowel sounds were normal, and gas out was normal, but gastric distension was noted. He complained of epigastric pain, indigestion, and early satiety for 1 week.

**Figure 1:**
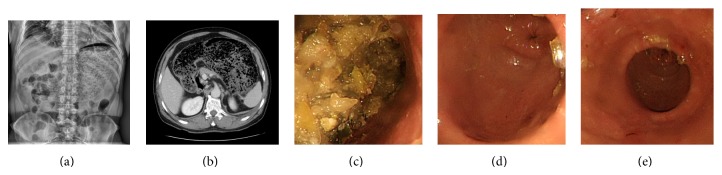
(a) Simple abdominal X-ray reveals a large amount of material in the stomach, suggesting severe gastric hypomotility. (b) Abdominopelvic computed tomography reveals a large amount of food material in the stomach, suggesting partial stricture in the postbulbar portion of the duodenum. (c) Endoscopic findings reveal large amounts of food stored in the gastric body and antrum. (d) The food was removed using a cap-fitted endoscope and a net over a period of days. (e) Peristaltic motion of the gastric antrum was normal and the pylorus opened and closed normally.

To prove gastroparesis related with RFCA, we used simple abdomen X-ray, abdominal pelvic CT, and gastroscopy as a diagnostic method. A simple abdominal X-ray revealed a large amount of material in the stomach, suggesting severe gastric hypomotility. Abdominal pelvic CT revealed large amount of food in the stomach (Figures 1(a) and 1(b)).

**Figure 2:**
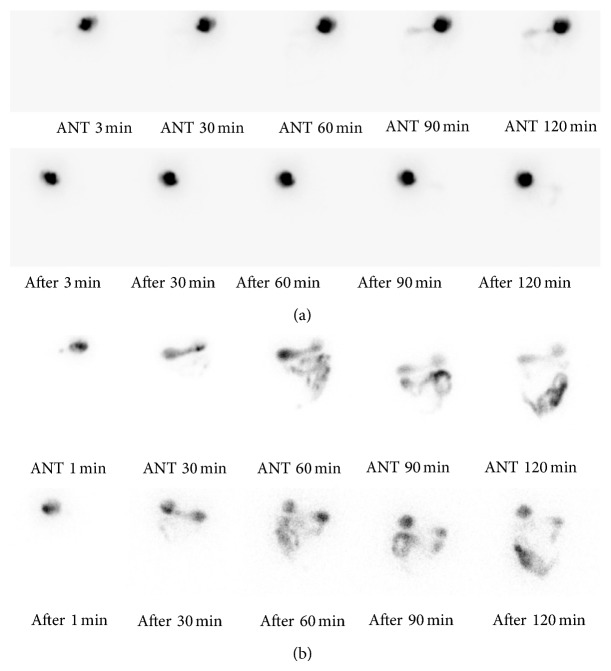
(a) Gastric emptying scan reveals delayed gastric emptying time with an almost flat time-activity curve. A large amount of contrast material remains in the 2-hour delayed image, suggesting severe gastric hypomotility. (b) Gastric emptying scan reveals delayed gastric emptying time, suggesting normal gastric motion.

Endoscopic findings revealed large amounts of food stored in the gastric body. Normal peristaltic motion of the gastric antrum and pylorus was observed (Figures 1(c), 1(d), and 1(e)). The food was removed using a cap-fitted endoscope and a net over a period of days. At the time of paralysis, specific lesions have not been found inside the gastrointestinal tract and biopsy was not performed. With suspected gastroparesis, he underwent a gastric emptying scan. The gastric emptying scan revealed delayed gastric emptying time with an almost flat time-activity curve. There still remained a large amount of contrast material in the 2-hour delayed image, suggesting severe gastric hypomotility (Figure 2(a)). He was treated with prokinetics (50 mg itopride three times daily) and antiulcer medication (100 mg rebamipide three times daily). After the start of treatment, digestive function and excretion function were improved by 20 percent in one week. Digestive function has been completely recovered in two months later. After normalization of gastrointestinal motility, there were no abnormal findings in the duodenum in follow-up gastroscopy examination (Figure 2(b)).

## 3. Discussion

The vagus nerve begins in the brain and extends into the abdomen. The vagus nerve is responsible for such varied tasks as heart rate, gastrointestinal peristalsis, sweating, and quite a few muscle movements in the mouth, including speech and keeping the larynx open for breathing. Digestive problems can sometimes occur as a result of vagus nerve damage. Persistent gastroparesis is often a symptom of nerve damage in this area. This is most commonly due to abnormalities or damage in the way the stomach and intestines contract. Decreased production of stomach acid is also a common symptom of this type of nerve damage [7].

Because the position of the vagus nerve is adjacent to the heart, gastroparesis due to the vagus nerve can occur due to various mechanical procedures such as postvagotomy syndrome for peptic ulcer or pancreatectomy due to pancreatic cancer. However, a vagus nerve injury due to electrical energy is very rare. Shah et al. reported four cases of acute delayed gastric emptying caused by vagus nerve injury after RF ablation for AF [2]. A case report of severe gastroparesis has been published recently [5]. Gastroparesis is observed as food retention via endoscopy and is supposedly caused by damage to the vagal nerve fibers surrounding the distal esophagus, as suggested in previous studies [5, 8]. Thus, delayed gastric emptying is considered a direct noncardiac complication of the ablation procedure.

We defined atrial fibrillation gut syndrome (AFGS) as symptoms of decreased gastrointestinal motility that occur after RFCA treatment. Our patient had no underlying disease other than AF, and digestive function was good. Because there was no underlying disease, we eliminated other causes for the gastroparesis. After treatment, severe abdominal distension findings revealed the gastroparesis findings, and AFGS was diagnosed. Although AFGS can occur based on treatment time, the vagus nerve tract, intensity of the electric force, treatment time, and RFCA site, it is reversible. RFCA treatment time is different according to the patient and center. In our center it took about 6 hours and it took up about 7 hours in this case. According to the existing literature, AFGS has been estimated by the treatment site and the amount of energy. Procedure time is also important, because a large amount of energy is transmitted in proportion to the time. In order to reduce the thermal injury to a minimum, we wrapped cooling pack to the portion of the neck in the procedure. We will prepare a prospective study on the effectiveness of the cooling pack to minimize the gastrointestinal paralysis on the above variables.

It took 6 months for the previous gastroparesis cases to recover to normal [5]. Our case was more serious, as the patient complained of terrible distension. However, gastric function completely recovered in 2 months with a rapid diagnosis, treatment, and exercise.

**Figure 3:**
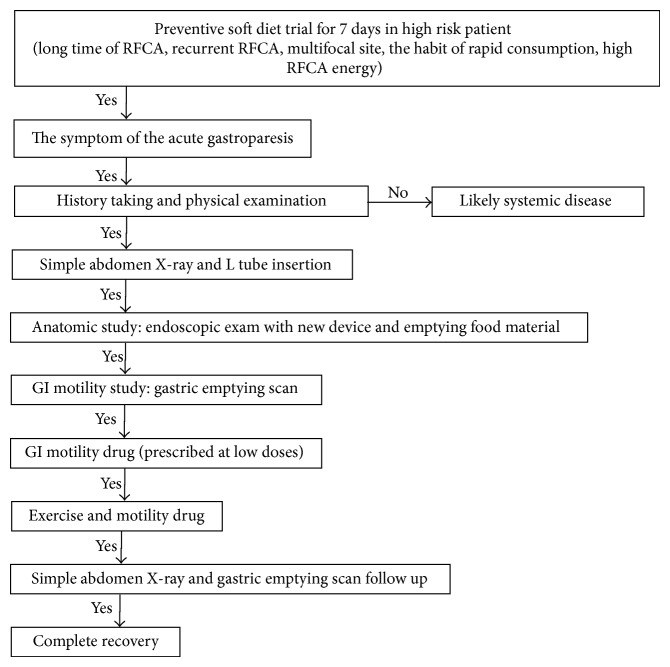
Flow chart of the diagnosis and treatment in our case and previous other cases.

It is believed that the recovery period can be reduced by rapid diagnosis and proper treatment and rehabilitation. We suggest a flow chart for diagnosis and treatment of AFGS based on the present case and previous cases. This flow chart can minimize the complications that occur after the operation in the AFGS high risk group. We recommend a soft diet in the high risk patient, as indicated in Figure 3, because it is easy to remove residual food material in cases of severe gastroparesis.

## 4. Conclusion

Although many complications can occur during RFCA, it is an absolutely necessary treatment for AF. AFGS is very unfamiliar complications to the cardiologists and gastroenterologists. It is difficult to predict AFGS on the basis of the patient symptoms. Our patient was the most serious case of AFGS that has been reported to date but shows that AFGS is fully reversible. In addition, this case presents the summary for diagnosing and treating AFGS to the RFCA practitioner. Considering these reversible and frequent AFGS complications, we think AFGS should be added to the common causative category of mechanical gastroparesis.

A feature of digestive tract paralysis due to vagus nerve damage is that movement of the wall of the digestive tract is paralyzed but movement of the pylorus is normal. Thus, the bowel is normal on a physical exam but retention of food material interferes with the pylorus. Removal of food is essential to resolve the abdominal distension and problem with the pylorus. According to our experience, endoscopic removal by colonoscopy is recommended for hardened food material.

**Figure 4:**
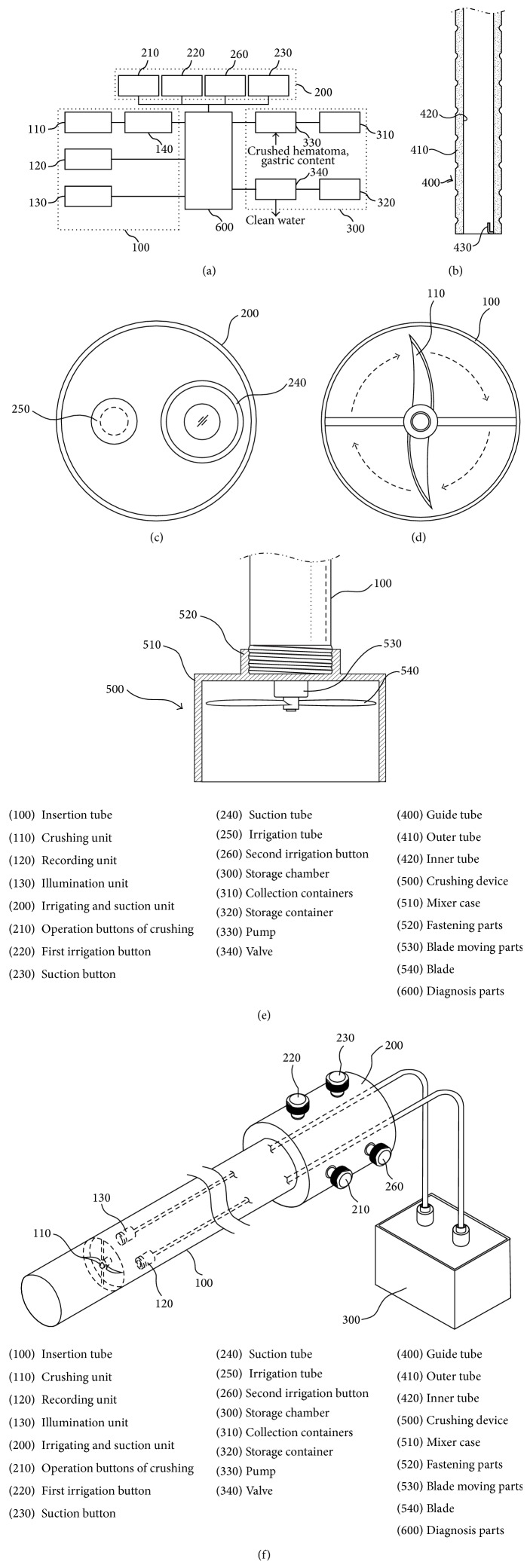
(a) Schematic control flow of the gastric content removing device; (b) endoscopic guide tube for evacuating gastric content; (c) irrigation and suction unit of the gastric contents; (d) mixer parts of crushing unit; (e) fastening parts of crushing unit; (f) appearance of the gastric content removing device.

Medical patent has been invented on the basis of the present case (patent number 10-2014-0102131, Republic of Korea) (Figure 4). Patent device is devised to eliminate gastric contents through the esophagus without surgery. By using the patent endoscope tube, sufficient internal space of the esophagus is ensured. Via a secure space, grinding endoscopic instrument is inserted and all of the gastric contents can be removed easily such as hematoma, foreign body, and food material. In future, fast diagnosis and treatment of AFGS will be possible. Various gastrointestinal foreign matter removals can be possible without surgery.
